# A phase I, randomized, double-blind, placebo-controlled, ascending single- and multiple-dose study of the pharmacokinetics, safety, and tolerability of oral ceftibuten in healthy adult subjects

**DOI:** 10.1128/aac.01099-23

**Published:** 2023-12-07

**Authors:** María Patricia Hernández-Mitre, Steven C. Wallis, Elizabeth E. Morgan, Michael N. Dudley, Jeffery S. Loutit, David C. Griffith, Jason A. Roberts

**Affiliations:** 1 UQ Centre for Clinical Research, Faculty of Medicine, The University of Queensland, Brisbane, Queensland, Australia; 2 Qpex Biopharma, Inc., San Diego, California, USA; 3 Herston Infectious Diseases Institute, Metro North Health, Brisbane, Queensland, Australia; 4 Departments of Intensive Care Medicine and Pharmacy, Royal Brisbane & Women’s Hospital, Brisbane, Queensland, Australia; 5 Division of Anaesthesiology Critical Care Emergency and Pain Medicine, Nîmes University Hospital, University of Montpellier, Nîmes, France; University of Pittsburgh, Pittsburgh, Pennsylvania, USA

**Keywords:** ceftibuten, phase I, pharmacokinetics, safety, tolerability

## Abstract

This was a phase I, randomized, double-blind, placebo-controlled, ascending single- and multiple-dose study of oral ceftibuten to describe the pharmacokinetics (PK) of *cis-*ceftibuten (administered form) and *trans-*ceftibuten (metabolite), and to describe safety and tolerability at higher than licensed doses. Subjects received single 400, 600, or 800 mg doses of ceftibuten on Days 1 and 4, followed by 7 days of twice-daily dosing from Days 4 to 10. Non-compartmental methods were used to describe parent drug and metabolite PK in plasma and urine. Dose proportionality was examined using *C*_max_, AUC_0–12_, and AUC_0–INF_. Accumulation was calculated as the ratio of AUC_0–12_ on Days 4 and 10. Adverse events (AEs) were monitored throughout the study. Following single ascending doses, mean *cis*- and *trans-*ceftibuten *C*_max_ were 17.6, 24.1, and 28.1 mg/L, and 1.1, 1.5, and 2.2 mg/L, respectively; *cis*-ceftibuten urinary recovery accounted for 64.3%–86.9% of the administered dose over 48 h. Following multiple ascending doses, mean *cis*- and *trans-*ceftibuten *C*_max_ were 21.7, 28.1, and 38.8 mg/L, and 1.4, 1.9, and 2.8 mg/L, respectively; *cis*-ceftibuten urinary recovery accounted for 72.2%–96.4% of the administered dose at steady state. The exposure of *cis-* and *trans-*ceftibuten increased proportionally with increasing doses. *Cis*- and *trans-*ceftibuten accumulation factor was 1.14–1.19 and 1.28–1.32. The most common gastrointestinal treatment emergent AEs were mild and resolved without intervention. Ceftibuten was well tolerated. Dose proportionality and accumulation of *cis*- and *trans*-ceftibuten were observed. These results support the ongoing development of ceftibuten at doses up to 800 mg twice-daily. (The study was registered at ClinicalTrials.gov under the identifier NCT03939429.)

## INTRODUCTION

The worldwide escalation of resistance to antibiotics among Gram-negative bacteria, particularly members of the ESKAPE group of pathogens (1), has resulted in a crisis for the treatment of many hospital-acquired and community-acquired infections. In particular, the increase in *Enterobacterales* expressing extended-spectrum β-lactamases (ESBLs) and carbapenemases that are resistant to all oral beta-lactams and fluoroquinolones in the community have resulted in many patients requiring hospital admission just for intravenous antibiotics to treat their infections. The associated cost for the healthcare system is high (2). Thus, new oral therapeutic options are needed (3).

Ceftibuten is a cephalosporin antibiotic approved in the USA (4) for acute exacerbations of chronic bronchitis, acute bacterial otitis media, and pharyngitis/tonsillitis at a dose of 400 mg once-daily for up to 10 days (5 – 7). The safety profile of ceftibuten is similar to other cephalosporin antibiotics with nausea (4%), headache (3%), diarrhea (3%), dyspepsia (2%) and dizziness, abdominal pain, and vomiting (all 1%) being the most common adverse reactions in the US Food and Drug Administration label (8).

The pharmacokinetics (PK) of ceftibuten has been characterized in single- and multiple-dose studies in the range of 25–800 mg (9 – 16). Previous studies have shown that ceftibuten is very well absorbed after oral administration of doses up to 400 mg, with a bioavailability between 75% and 90% (6, 7, 13, 16). Maximum plasma concentrations are reached within 2–3 h (9, 12, 14). The elimination of the drug is primarily renal (~70%–80% of total clearance) (6, 9, 16), with a half-life of ~2–3 h (10 – 16). The metabolism of ceftibuten is minimal, involving isomerization to *trans*-ceftibuten (17), which exhibits ~8-fold lower antimicrobial potency compared to *cis-*ceftibuten (9, 11, 13, 16). Approximately 60%–70% of an administered dose of ceftibuten is recovered in the urine as unchanged drug (*cis-*ceftibuten), and a further 10%–20% as *trans-*ceftibuten (6, 12, 13).

Qpex Biopharma (San Diego, CA, USA) is developing a fixed combination antibiotic of ceftibuten (QPX2015) with a new β-lactamase inhibitor (xeruborbactam, formerly QPX7728). Xeruborbactam can be given both intravenously and orally (in prodrug form), and has inhibitory activity against ESBLs and carbapenemases, including both serine and metallo-β-lactamases (18, 19). To maximize the efficacy of ceftibuten in more serious infections due to Gram-negative bacilli, higher doses of ceftibuten than those currently approved may be useful.

The aim of this study is to describe the PK, safety and tolerability of single and multiple oral doses of ceftibuten administered to healthy adult subjects at doses higher than the 400 mg once-daily approved in the USA.

## RESULTS

In total, 40 subjects were included in the study; 32 subjects received ceftibuten and 8 received placebo. One subject withdrew prior study completion and did not provide data for Day 10 of the 800 mg dose (*n* = 15). The demographics for all subjects are summarized in Table 1. Overall, demographics were broadly consistent between the pooled ceftibuten and pooled placebo groups. As expected in these healthy volunteers, the mean baseline estimated glomerular filtration rate across all groups was 90 mL/min/1.73 m^2^ with no difference between groups.

**TABLE 1:** Demographics of the study population^*a*^

	Ceftibuten (400 mg*; N* = 8)	Ceftibuten (600 mg*; N* = 8)	Ceftibuten (800 mg*; N* = 16)	Pooled ceftibuten (*N* = 32)	Pooled placebo (*N* = 8)	Overall (*N* = 40)
Age (years)	34.3 ± 9.5	30 ± 11.6	31.5 ± 10	31.8 ± 10.1	31.9 ± 11.7	31.8 ± 10.3
Male, *n* (%)	7 (87.5)	8 (100)	15 (93.8)	30 (93.8)	7 (87.5)	37 (92.5)
Height (cm)	180.5 ± 9.2	179 ± 5.2	174.8 ± 8.2	177.3 ± 8	175.3 ± 11.3	176.9 ± 8.7
Weight (kg)	77.8 ± 10.1	77.3 ± 9.3	79.1 ± 9.7	78.3 ± 9.4	72 ± 3.6	77 ± 8.9
BMI (kg/m^2^)	23.8 ± 1.2	24.2 ± 3	25.9 ± 2.6	24.9 ± 2.6	23.7 ± 3	24.7 ± 2.7
White, *n* (%)	8 (100)	8 (100)	9 (56.3)	25 (78.1)	6 (75)	31 (77.5)

^*a*^ Statistics are presented as mean ± SD for continuous variables and count (%) for categorical variables. *N*, number of subjects; BMI, body mass index.

### Single dose plasma PK of *cis-* and *trans-*ceftibuten

**Fig 1:**
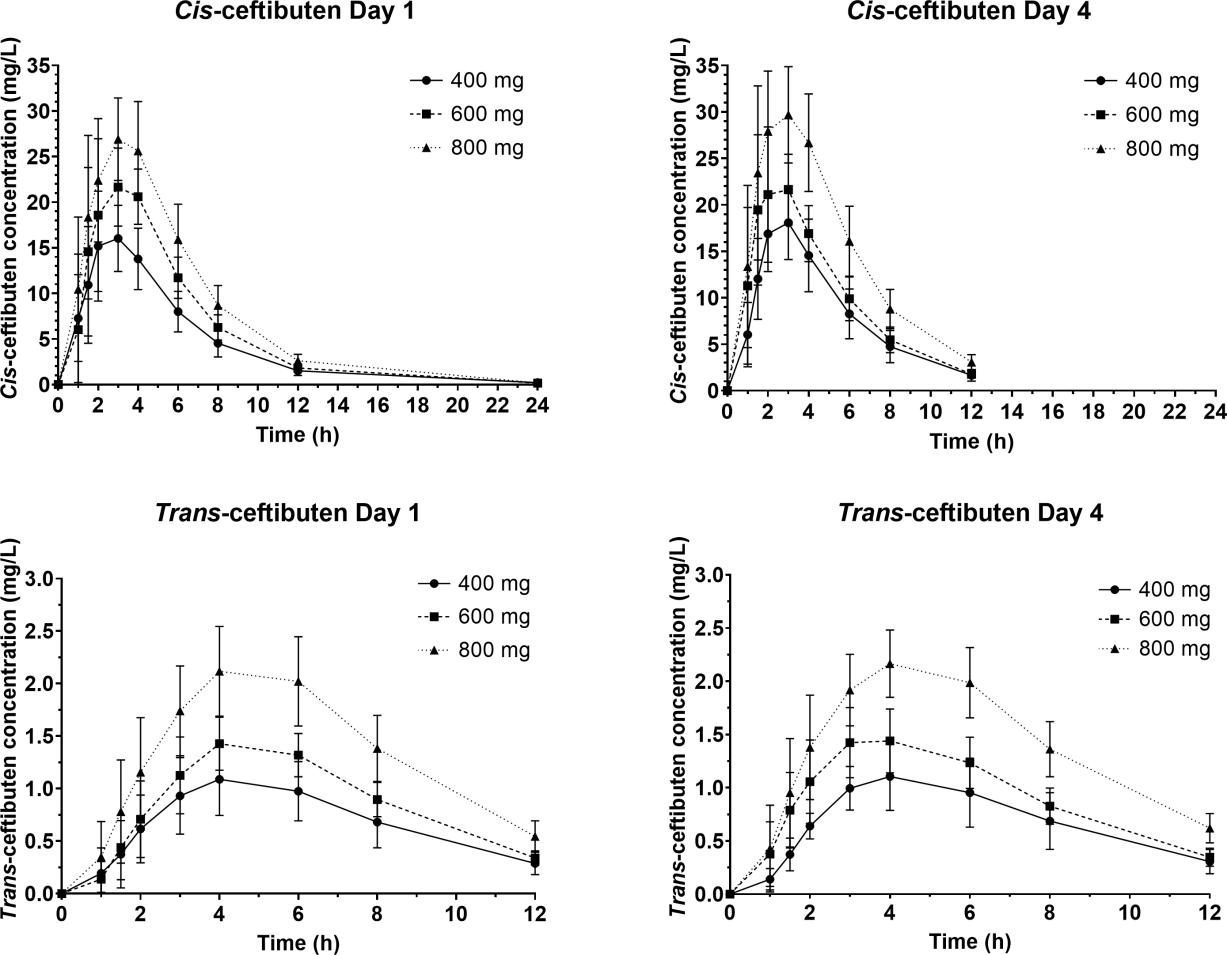
*Cis*- and *trans*-ceftibuten plasma concentrations (mean ± SD) on Days 1 and 4 after single oral administration in healthy adult subjects.

*Cis-* and *trans-*ceftibuten plasma PK profiles on Days 1 and 4 after single oral doses of 400, 600, and 800 mg are shown in Fig. 1.

Both *cis*- and *trans-*ceftibuten exhibited a dose-dependent increase in their respective mean maximum observed plasma concentration (*C*_max_) values. The time of maximum observed plasma concentration (*T*_max_) ranged from 2.6 to 3 h for *cis-*ceftibuten and from 4.4 to 4.8 h for *trans*-ceftibuten. Additionally, both *cis-* and *trans-*ceftibuten showed a dose-related rise in the mean area under the plasma concentration-time curve from 0 to 12 h (AUC_0–12_) and from 0 extrapolated to time infinity (AUC_0–INF_). The mean terminal half-life (*t*_1/2_) remained consistent across the studied dose range, with values ranging from 2.3 to 2.7 h for *cis-*ceftibuten and 3.1–3.2 h for *trans-*ceftibuten. *Cis-*ceftibuten mean apparent volume of distribution (Vz/*F*) ranged from 15.8 to 18.9 L, and mean apparent clearance (CL/*F*) ranged from 4.4 to 4.9 L/h. *Cis-* and *trans-*ceftibuten plasma PK parameters on Day 4 were similar to those on Day 1 (Table 2).

**TABLE 2:** *Cis-* and *trans-*ceftibuten plasma and urine pharmacokinetic parameters (mean ± SD) by dosing group after single oral doses on Days 1 and 4^*a*^

	Day 1			Day 4		
	400 mg	600 mg	800 mg	400 mg	600 mg	800 mg
Cis-ceftibuten						
*N*	8	8	16	8	8	16
*C*_max_ (mg/L)	17.60 ± 3.95	24.14 ± 4.77	28.11 ± 4.00	18.45 ± 3.54	23.99 ± 4.84	32.04 ± 4.69
*T*_max_ (h)	2.63 ± 1.06	2.81 ± 0.92	2.97 ± 0.92	2.75 ± 0.46	2.38 ± 0.88	2.63 ± 0.87
AUC_0–12_ (mg × h/L)	89.59 ± 20.34	120.86 ± 19.19	158.32 ± 27.28	95.08 ± 21.87	118.11 ± 18.59	172.46 ± 25.77
AUC_0–INF_ (mg × h/L)	95.28 ± 21.97	127.28 ± 21.34	169.28 ± 29.28	101.30 ± 24.25	124.21 ± 19.95	183.27 ± 27.32
*t*_1/2_ (h)	2.53 ± 0.39	2.34 ± 0.33	2.68 ± 0.29	2.56 ± 0.31	2.40 ± 0.18	2.50 ± 0.22
Vz/*F* (L)	15.79 ± 2.98	16.06 ± 1.78	18.87 ± 4.37	15.13 ± 2.94	17.05 ± 2.47	16.14 ± 3.12
CL/*F* (L/h)	4.38 ± 0.93	4.82 ± 0.75	4.88 ± 1.01	4.14 ± 0.96	4.94 ± 0.81	4.45 ± 0.63
Ae (mg)	332.59 ± 200.78	521.11 ± 71.96	514.69 ± 103.21	259.98 ± 70.77	461.29 ± 45.03	475.89 ± 104.20
fe (%)	83.15 ± 50.19	86.85 ± 11.99	64.34 ± 12.90	64.99 ± 17.69	76.88 ± 7.50	59.49 ± 13.02
CL_R_ (L/h)	3.52 ± 1.91	4.16 ± 0.68	3.11 ± 0.72	–	–	–
Trans-ceftibuten						
*N*	8	8	16	8	8	16
*C*_max_ (mg/L)	1.10 ± 0.34	1.46 ± 0.24	2.19 ± 0.41	1.11 ± 0.32	1.49 ± 0.28	2.20 ± 0.31
*T*_max_ (h)	4.38 ± 1.06	4.75 ± 1.04	4.69 ± 1.08	3.88 ± 0.35	3.75 ± 1.04	4.19 ± 1.05
AUC_0–12_ (mg × h/L)	7.87 ± 2.41	9.96 ± 1.71	15.47 ± 2.93	7.90 ± 2.27	10.53 ± 1.92	16.17 ± 2.29
AUC_0–INF_ (mg × h/L)	9.11 ± 3.37	11.56 ± 2.36	18.20 ± 3.40	9.56 ± 2.92	12.50 ± 2.37	19.20 ± 2.10
*t*_1/2_ (h)	3.17 ± 0.23	3.07 ± 0.29	3.12 ± 0.45	3.63 ± 0.51	3.29 ± 0.26	3.55 ± 0.54
Ae (mg)	43.77 ± 19.99	74.96 ± 13.06	92.66 ± 21.93	37.36 ± 10.21	64.44 ± 14.64	78.91 ± 22.54
fe (%)	10.94 ± 5.00	12.49 ± 2.18	11.58 ± 2.74	9.34 ± 2.55	10.74 ± 2.44	9.86 ± 2.82
CL_R_ (L/h)	5.33 ± 2.95	6.34 ± 1.36	4.83 ± 1.03	–^*b*^	–	–
Total ceftibuten						
*N*	8	8	16	8	8	16
Ae (mg)	376.37 ± 219.96	596.07 ± 82.95	607.35 ± 119.00	297.34 ± 78.10	525.72 ± 54.50	554.80 ± 119.55
fe (%)	94.09 ± 54.99	99.34 ± 13.82	75.92 ± 14.88	74.33 ± 19.52	87.62 ± 9.08	69.35 ± 14.94
CL_R_ (L/h)	3.96 ± 2.19	4.42 ± 0.86	3.24 ± 0.86	–	–	–

^*a*^ *N*, number of subjects; *C*_max_, maximum observed plasma concentration; *T*_max_, time of maximum observed plasma concentration; AUC_0–12_, area under the plasma concentration-time curve from 0 to 12 h; AUC_0–INF_, area under the plasma concentration-time curve from 0 extrapolated to time infinity; *t*_1/2_, terminal half-life; Vz/*F*, apparent volume of distribution; CL/*F*, apparent clearance; Ae, amount of drug excreted in the urine (over 48 h on Day 1 and 12 h on Day 4); fe, fraction of the administered dose excreted in the urine (over 48 h on Day 1 and 12 h on Day 4); CL_R_, renal clearance (Ae_0–48_/AUC_0–INF_).

^*b*^ "–” no data available.

### Single dose urine PK of *cis-, trans-*, and total ceftibuten

*Cis-*ceftibuten mean amount of drug excreted in the urine (Ae) over 48 h ranged from 332.6 mg for the 400 mg dose to 521.1 mg for the 600 mg dose. The mean fraction of the administered dose excreted in the urine (fe) varied from 64.3% to 86.9%. Mean renal clearance (CL_R_) ranged from 3.1 to 4.2 L/h (Table 2).

*Trans-*ceftibuten mean Ae over 48 h ranged from 43.8 mg for the 400 mg dose to 92.7 mg for the 800 mg dose. The mean fe varied from 10.9% to 12.5%. Mean CL_R_ ranged from 4.8 to 6.3 L/h (Table 2).

Total ceftibuten mean Ae over 48 h ranged from 376.4 mg for the 400 mg dose to 607.4 mg for the 800 mg dose. The mean fe varied from 75.9% to 99.3%. Mean CL_R_ ranged from 3.2 to 4.4 L/h (Table 2). In general, renal excretion is a significant pathway of ceftibuten elimination.

### Multiple dose plasma PK of *cis-* and *trans-*ceftibuten

**Fig 2:**
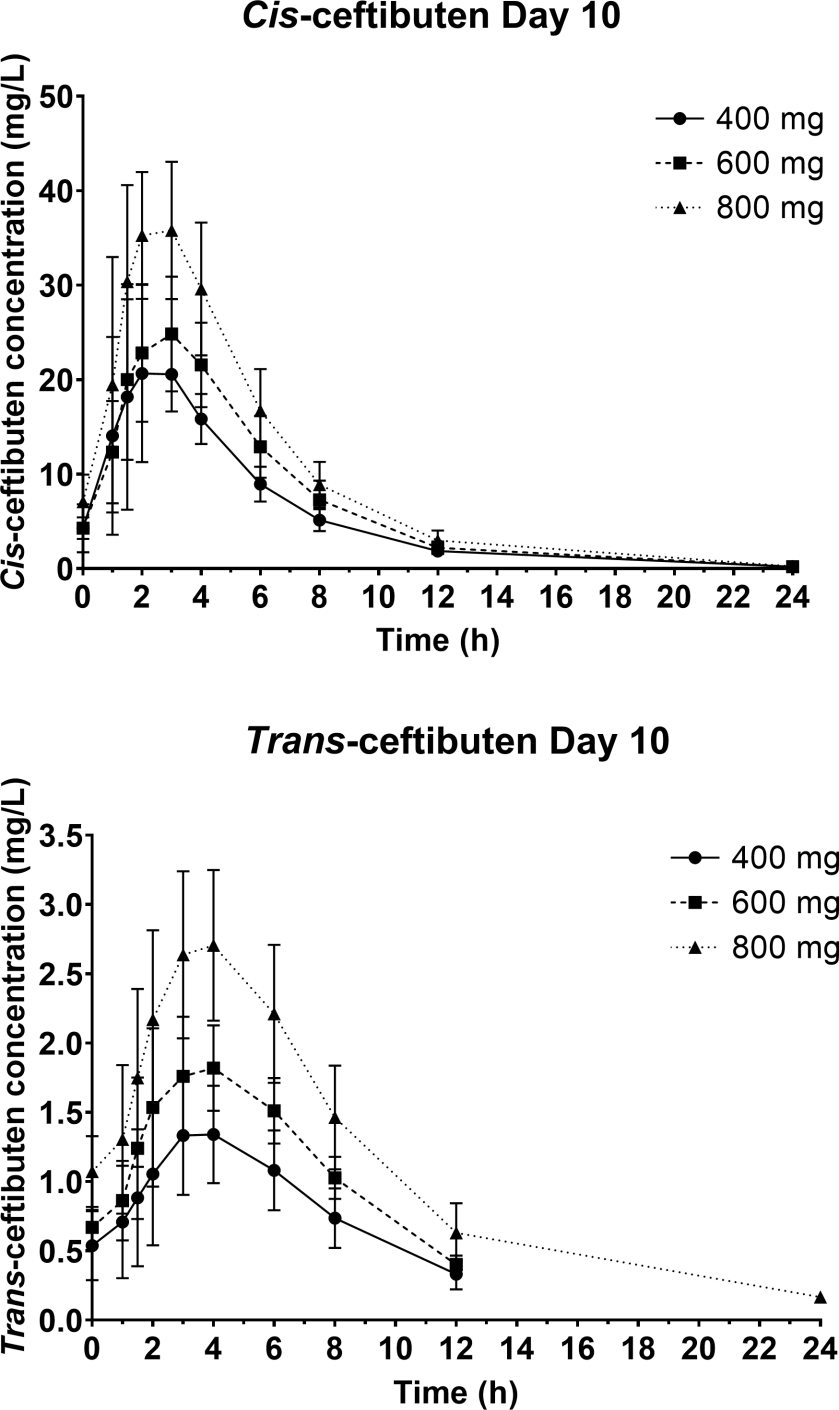
Washout *cis*- and *trans*-ceftibuten plasma concentrations (mean ± SD) on Day 10 after 7 days of twice-daily oral administration in healthy adult subjects.

*Cis-* and *trans-*ceftibuten plasma PK profiles after 7 days (Day 10) of ceftibuten administered twice-daily at doses of 400, 600, and 800 mg are shown in Fig. 2.

Both *cis*- and *trans-*ceftibuten exhibited a dose-dependent increase in their respective *C*_max_ values. *T*_max_ ranged from 2.3 to 2.4 h for *cis-*ceftibuten and from 3.3 to 3.5 h for *trans*-ceftibuten. Additionally, both *cis-* and *trans-*ceftibuten showed a dose-related rise in AUC_0–12_ and AUC_0–INF_ (washout) values. The mean *t*_1/2_ remained consistent across the studied dose range, with values ranging from 2.7 to 3.1 h for *cis-*ceftibuten and 3.0–3.5 h for *trans-*ceftibuten. *Cis-*ceftibuten mean apparent volume of distribution (Vz/*F*) ranged from 16.8 to 18.0 L, and mean apparent clearance (CL/*F*) ranged from 3.9 to 4.3 L/h (Table 3).

**TABLE 3:** *Cis-* and *trans-*ceftibuten plasma and urine pharmacokinetic parameters (mean ± SD) by dosing group after multiple doses on Day 10^*a*^

	Day 10		
	400 mg	600 mg	800 mg
Cis-ceftibuten			
*N*	8	8	15
*C*_max_ (mg/L)	21.65 ± 5.68	28.06 ± 4.59	38.76 ± 7.18
*T*_max_ (h)	2.31 ± 0.80	2.31 ± 0.92	2.43 ± 0.82
AUC_0–12_ (mg × h/L)	109.79 ± 29.86	140.18 ± 17.56	197.84 ± 29.52
AUC_0–INF_ (mg × h/L)	118.58 ± 32.00	148.91 ± 19.69	212.26 ± 32.71
*t*_1/2_ (h)	3.12 ± 0.48	2.71 ± 0.36	3.02 ± 0.29
Vz/*F* (L)	17.26 ± 4.66	16.77 ± 1.74	17.96 ± 3.15
CL/*F* (L/h)	3.85 ± 0.89	4.34 ± 0.54	4.13 ± 0.67
Ae_0–12_ (mg)	322.75 ± 87.91	578.14 ± 21.37	577.91 ± 144.83
fe_0–12_ (%)	80.69 ± 21.98	96.36 ± 3.56	72.24 ± 18.10
CL_R_ (L/h)	3.08 ± 1.06	4.18 ± 0.52	2.97 ± 0.80
Trans-ceftibuten			
*N*	8	8	15
*C*_max_ (mg/L)	1.38 ± 0.39	1.89 ± 0.36	2.79 ± 0.56
*T*_max_ (h)	3.50 ± 0.53	3.50 ± 1.31	3.27 ± 0.80
AUC_0–12_ (mg × h/L)	10.27 ± 3.23	13.90 ± 2.30	20.45 ± 4.00
AUC_0–INF_ (mg × h/L)	11.99 ± 3.83	16.06 ± 2.30	23.58 ± 4.98
*t*_1/2_ (h)	3.50 ± 0.44	3.05 ± 0.28	3.31 ± 0.51
Ae_0–12_ (mg)	48.22 ± 10.96	86.51 ± 6.02	98.28 ± 29.84
fe_0–12_ (%)	12.06 ± 2.74	14.42 ± 1.00	12.29 ± 3.73
CL_R_ (L/h)	5.06 ± 1.69	6.33 ± 0.85	4.88 ± 1.45
Total ceftibuten			
*N*	8	8	15
Ae_0–12_ (mg)	370.98 ± 98.05	664.65 ± 21.95	676.19 ± 172.37
fe_0–12_ (%)	92.74 ± 24.51	110.77 ± 3.66	84.52 ± 21.55
CL_R_ (L/h)	3.24 ± 1.10	4.37 ± 0.54	3.15 ± 0.84

^*a*^ *N*, number of subjects; *C*_max_, maximum observed plasma concentration; *T*_max_, time of maximum observed plasma concentration; AUC_0–12_, area under the plasma concentration-time curve from 0 to 12 h; AUC_0–INF_, area under the plasma concentration-time curve from 0 extrapolated to time infinity; *t*_1/2_, terminal half-life; Vz/*F*, apparent volume of distribution; CL/*F*, apparent clearance; Ae**_0–12_**, amount of drug excreted in the urine at steady state; fe**_0–12_**, fraction of the administered dose excreted in the urine at steady state; CL_R_, renal clearance (Ae_0–12_/AUC_0–12_).

### Multiple dose urine PK of *cis-, trans-,* and total ceftibuten

*Cis-*ceftibuten mean Ae at steady state (Ae_0–12_) ranged from 322.8 mg for the 400 mg dose to 578.1 mg for the 600 mg dose. The mean fe at steady state (fe_0–12_) varied from 72.2% to 96.4%. Mean CL_R_ ranged from 3.0 to 4.2 L/h (Table 3).

*Trans-*ceftibuten mean Ae_0–12_ ranged from 48.2 mg for the 400 mg dose to 98.3 mg for the 800 mg dose. The mean fe_0–12_ varied from 12.1% to 14.4%. Mean CL_R_ ranged from 4.9 to 6.3 L/h (Table 3).

Total ceftibuten mean Ae_0–12_ ranged from 371.0 mg for the 400 mg dose to 676.2 mg for the 800 mg dose. The mean fe_0–12_ varied from 84.5% to 110.8%. Mean CL_R_ ranged from 3.2 to 4.4 L/h (Table 3).

### Dose proportionality

The exposure (AUC_0–12_) of *cis-*ceftibuten increased with increasing doses. Linear regression analysis of the data from Day 1 (*y =* 0.1718*x* + 19.83; *r* = 0.9986), Day 4 (*y* = 0.1966*x +* 11.01*; r* = 0.9726), and Day 10 (*y* = 0.2201*x* + 17.20; *r =* 0.9844) showed that increases were dose proportional. The analysis of variance (ANOVA) confirmed the dose proportionality of *cis-*ceftibuten since no statistical differences were found for AUC_0–12_ and AUC_0–INF_ on Days 1, 4, and 10 (*P* > 0.05). Dose proportionality was also assessed using *C*_max_ after multiple doses (Day 10) and for a single dose (Day 4). However, a statistically significant difference was seen only between the 400 and 800 mg mean *C*_max_ on Day 1 for a single dose (*P* = 0.024; Tukey–Kramer post hoc test).

The AUC_0–12_ of *trans*-ceftibuten also increased with increasing doses. Linear regression analysis of the data from Day 1 (*y =* 0.019*x* − 0.3; *r =* 0.9679), Day 4 (*y =* 0.0202*x* − 0.6267; *r =* 0.9805), and Day 10 (*y* = 0.0254*x* − 0.3967; *r* = 0.9866) showed that increases were dose proportional. The ANOVA confirmed the dose proportionality of *trans*-ceftibuten since no statistically significant differences were found between the mean values of *C*_max_, AUC_0–12_, and AUC_0–INF_ on the three sampling days (*P* > 0.05).

### Accumulation

*Cis-*ceftibuten mean accumulation ratios were 1.15 ± 0.07, 1.19 ± 0.07, and 1.14 ± 0.17 for the 400, 600, and 800 mg doses, respectively. *Trans-*ceftibuten mean accumulation ratios were 1.30 ± 0.12, 1.32 ± 0.07, and 1.28 ± 0.21 for the 400, 600, and 800 mg doses, respectively. These values indicate *cis-* and *trans-*ceftibuten accumulation over 7 days of multiple dosing.

### Safety and tolerability

There were no serious adverse events (AEs), deaths, or other significant AEs reported in the study.

In the single ascending dose (SAD) period of the study, 18 (56.3%) of 32 subjects receiving ceftibuten reported a treatment emergent adverse event (TEAE), and 4 (50%) of 8 subjects reported five events in the pooled placebo group. Overall, the most frequently reported TEAEs in the 32 subjects in the total ceftibuten group were: headache, 9 events reported in 7 (21.9%) subjects; dysgeusia, 4 events reported in 3 (9.4%) subjects; and nausea, 4 events reported in 4 (12.5%) subjects. All TEAEs were mild or moderate in severity. No subjects discontinued from the study during the SAD period of the study.

In the multiple ascending dose (MAD) period of the study, 22 (68.8%) of 32 subjects that received ceftibuten reported 65 TEAEs compared to 3 (37.5%) of 8 subjects that reported 9 events in the pooled placebo group. Overall, the most frequently reported TEAEs in the 32 subjects that received ceftibuten were: headache, 14 events reported in 8 (25%) subjects; nausea, abdominal pain and diarrhea, each with 4 events reported in 4 (12.5%) subjects; and upper abdominal pain and dysgeusia, each with 3 events reported in 3 (9.4%) subjects (Table 4). The majority of TEAEs reported during the MAD period of the study were assessed as mild in severity, transient, and resolved while subjects were still receiving study drug. The incidence, but not severity, of gastrointestinal TEAEs increased with increasing dose. One subject in the ceftibuten treatment group (800 mg) discontinued from the study due to persistent nausea.

**TABLE 4:** Treatment emergent adverse events observed in three or more subjects receiving ceftibuten during the multiple ascending dose period of the study^*a*^

	*N* (%) *E*				
	Ceftibuten 400 mg BID (*N* = 8)	Ceftibuten 600 mg BID (*N* = 8)	Ceftibuten 800 mg BID (*N* = 16)	Pooled ceftibuten BID (*N* = 32)	Pooled placebo BID (*N* = 8)
Subjects with at least 1 TEAE	6 (75.0%) 13	5 (62.5%) 12	11 (68.8%) 40	22 (68.8%) 65	3 (37.5%) 9
Headache	1 (12.5%) 1	1 (12.5%) 1	6 (37.5%) 12	8 (25.0%) 14	2 (25.0%) 4
Nausea	0	0	4 (25.0%) 4	4 (12.5%) 4	1 (12.5%) 1
Abdominal pain	1 (12.5%) 1	0	3 (18.8%) 3	4 (12.5%) 4	0
Diarrhea	0	0	4 (25.0%) 4	4 (12.5%) 4	0
Upper abdominal pain	0	0	3 (18.8%) 3	3 (9.4%) 3	0
Dysgeusia	1 (12.5%) 1	2 (25.0%) 2	0	3 (9.4%) 3	0

^*a*^ *N*, number of subjects; *E*, number of events; BID, twice-daily; TEAE, treatment emergent adverse event.

Alanine aminotransferase (ALT) elevations were all mild in severity, asymptomatic, not associated with bilirubin elevations, and resolved following cessation of treatment and were observed in 1 (12.5%) of 8 subjects in the 400 mg ceftibuten group, 3 (37.5%) of 8 subjects in the 600 mg group, and 6 (37.5%) of 16 subjects in the 800 mg ceftibuten group.

No clinically significant trends were observed in other chemistry data, hematology, coagulation, urinalysis, vital signs, or electrocardiograms.

## DISCUSSION

The data described in this study after single and multiple twice-daily oral ceftibuten administered to healthy adult subjects at doses of 400 mg is consistent with previously published data for *C*_max_, *T*_max_, *t*_1/2_, total body clearance, urinary recovery, and CL_R_ of both *cis-* and *trans-*ceftibuten (12, 14, 15). However, in this study, the mean *cis*-ceftibuten AUC_0–12_ values were higher than those previously reported [89.6 vs 83.8 and 79.2 mg × h/L after a single 400 mg dose (12, 15) and 109.8 vs 97.1 mg × h/L after multiple twice-daily 400 mg doses (12)]. Furthermore, following the administration of a single oral dose of 800 mg of ceftibuten, the mean *cis-* and *trans-*ceftibuten *C*_max_ (28.1 and 2.2 mg/L, respectively) and AUC_0–12_ (158.3 and 15.5 mg × h/L) were also higher than the values reported by Lin et al. (15) (*C*_max_ of 23.3 and 1.6 mg/L, and AUC_0–12_ of 118.0 and 10.4 mg × h/L for *cis-* and *trans-*ceftibuten, respectively). Differences between the Bressolle et al. (12), Lin et al. (15), and the present study could be attributed to the age demographics of the participants in the respective studies. Subjects were generally younger in the Bressolle et al. (12) (*n* = 12, age range 18–26, mean age ± SD = 23.7 ± 1.9 years) and the Lin et al. (15) (*n* = 4, age range 19–38, mean age not provided) studies compared to those in our study (*n* = 8, age range 18–55, mean age ± SD = 34.3 ± 9.5 years).

Previous studies have explored the PK of ceftibuten after single oral doses of 25, 50, 100, 200, 300, 400, and 800 mg (9, 11 – 15), and multiple twice-daily oral doses in the range of 100–400 mg (9, 10, 12, 13).

To the best of our knowledge, this is the first study to report *cis-* and *trans-*ceftibuten plasma and urine PK following a single oral dose of 600 mg and multiple twice-daily oral doses of 600 and 800 mg. Our findings revealed measurable *cis*- and *trans*-ceftibuten concentrations in urine, and we observed dose proportional plasma PK. Previous studies of oral ceftibuten in humans demonstrated that plasma exposures of ceftibuten increased in proportion to the administered dose for doses ranging from 25 to 200 mg (9). However, only Lin et al. (15) evaluated dose proportionality at higher doses of 200, 400, and 800 mg using similar statistical approaches to those used in this study. The authors concluded that after the administration of 200 and 400 mg of ceftibuten, plasma exposures increased in proportion to the administered dose, whereas following the 800 mg dose, exposures were lower than expected and not concluded to be dose proportional. In the present study, dose proportionality in plasma for 400, 600, and 800 mg was observed. Interestingly, although the *C*_max_ after a single dose on Day 1 was not dose proportional between 400 and 800 mg, dose proportionality was seen after a single dose on Day 4 and after multiple doses (Day 10).

In our study, we observed a higher ceftibuten exposure than reported by Lin et al. (15) with a single 800 mg dose. The values we measured meant that we could conclude the presence of dose proportionality, a conclusion that Lin et al. (15) did not reach. There are notable differences between the two studies. Lin et al. (15) assessed dose proportionality after single doses of 200, 400, and 800 mg, whereas in our study, dose proportionality was assessed more comprehensively using doses of 400, 600, and 800 mg on Days 1, 4, and 10. Additionally, Lin et al. (15) evaluated dose proportionality based on a 200 mg dose, which is lower than the 400 mg dosage employed in our study. Furthermore, the formulation of the materials used by Lin et al. (15) is unknown. In contrast, we utilized commercially available 200 mg ceftibuten capsules, and all subjects were dosed in a fasting state. These differences may have contributed to the varying results observed.

The accumulation ratios of *cis*- and *trans*-ceftibuten following multiple doses of 400, 600, and 800 mg twice-daily of 1.14–1.19 and 1.28–1.32, are similar to those previously reported for 400 mg twice-daily of 1.24 (12) and 1.17 (12), respectively; the *cis*-ceftibuten accumulation is also in line with the ratio of 1.13 reported following multiple daily oral ceftibuten doses of 400 mg (14), and 1.2 after multiple twice-daily 200 mg doses (13). These data provide reassurance of the consistency of *cis*- and *trans*-ceftibuten PK in healthy adult volunteers.

The present study found that ceftibuten was well tolerated when administered in all studied single and multiple doses. There were no serious, severe, life-threatening TEAEs, or TEAEs resulting in death reported throughout the study (SAD and MAD periods).

In the SAD period of the study, at ceftibuten doses up to 800 mg, the majority of TEAEs were mild in severity and there was no evidence of increasing incidence or severity of TEAEs with increasing dose. In the MAD period of the study, at ceftibuten doses up to 800 mg twice-daily for 7 days, the frequency of TEAEs was similar among all dose levels; however, the number of subjects reporting TEAEs in the total ceftibuten group was approximately twice that of the pooled placebo group. Mild ALT increases occurred in all cohorts with an increase in the incidence with increasing doses of ceftibuten. Approximately 30% of subjects in the 600 and 800 mg cohorts had an increase in their ALT, all of which were less than three times the upper limit of normal of 51 IU/L. ALT increases were initially observed 4–5 days after initiation of dosing and were maximal 3 days after the completion of dosing. All increases were asymptomatic, resolved spontaneously over the follow-up period and were not associated with bilirubin or alkaline phosphatase increases. No subjects discontinued dosing due to ALT increases. Oral cephalosporins have been linked to slight elevations in ALT and alkaline phosphatase levels, which are typically mild, temporary, and not accompanied by any symptoms or progression to more severe liver injury. The reported frequency of these elevations can reach up to 11%, although this rate varies based on factors such as the frequency of monitoring, duration of treatment, and nature and severity of the underlying disease (20).

Overall, the most commonly reported TEAEs were headache, nausea, diarrhea, and abdominal pain, the majority of which were mild in severity. The TEAEs observed in this study are similar to those reported with ceftibuten (8, 20). However, the incidence of headache and gastrointestinal TEAEs was higher than previously reported in the ceftibuten 800 mg twice-daily group. These SAD and MAD data are reassuring about the tolerability profile of ceftibuten at therapeutic doses, although future larger studies may provide further specific data.

Based on recent publications, the pharmacodynamic target for ceftibuten is free ceftibuten concentrations above the minimum inhibitory concentration (MIC) for 60%–67% of the dosing interval (21, 22). Based on this PK/PD target, 400 mg twice-daily should adequately cover MICs up to 1 mg/L and for 800 mg twice-daily, MICs up to 4 mg/L.

### Conclusion

The plasma and urine PK of *cis*-ceftibuten and its *trans* metabolite were investigated in healthy adult subjects following single and multiple oral doses higher than the 400 mg once-daily approved in the USA. CL_R_ was the significant pathway for the total CL of ceftibuten, which was mainly recovered as *cis*-ceftibuten. Dose proportionality was seen following single and multiple doses of 400, 600, and 800 mg. There is little drug accumulation of *cis*- and *trans*-ceftibuten during multiple dosing. Overall, the safety and PK data from this study supports the ongoing development of ceftibuten for single and multiple doses up to and including 800 mg twice-daily.

## MATERIALS AND METHODS

The ceftibuten used in the trial was sourced from Shionogi & Co, Ltd (Osaka, Japan); each capsule contained 200 mg of ceftibuten.

This research adhered to the ethical standards outlined in the Declaration of Helsinki (Ethical Principles for Medical Research Involving Human Subjects) and the National Health and Medical Research Council (NHMRC) (23) (updated 2018) (23). The protocol was approved on 5 April 2019 by the Bellberry Human Research Ethics Committee (HREC). The study was registered on ClinicalTrials.gov under the identifier: NCT03939429.

### Study design

This was a double-blind, randomized, placebo-controlled, and sequential ascending single- and multiple-dose study of oral ceftibuten.

Three doses were studied. The 400 and 600 mg dosing groups consisted of 10 subjects (8 active drug and 2 placebo), while the 800 mg group consisted of 20 subjects (16 active drug and 4 placebo). Within each group, subjects received a single oral dose on Days 1 and 4 (SAD); then, after the first dose on Day 4, 12-hourly doses were administered for 7 days (Days 4–10) (MAD).

#### Blinding

Breaking of the blind prior to study completion was expressly forbidden except in the event of a medical emergency where the identity of the drug had to be known to properly treat the subject or to assess the stopping rules.

#### Randomization

In each cohort, subjects who had signed informed consent and met all eligibility criteria were randomized to receive active treatment or placebo according to their assigned unique subject randomization number. The randomization scheme was devised by an unblinded statistician (who had no other involvement in the study and was considered blinded) using statistical computer software suitable for generating randomizations, such as SAS PROC PLAN. The randomization codes were made available in a secure electronic format only to the site pharmacy staff administering the study drug who were not involved in any other aspect of the study. The codes were not made available to the Sponsor, study subjects, or staff members responsible for the monitoring and evaluation of safety assessments.

#### Placebo

Matched placebo capsules containing microcrystalline cellulose for oral administration were used. Bottles containing placebo were to be stored refrigerated at 2°C–8°C and handled as described in the study Pharmacy Manual.

### Subjects

Healthy adult subjects aged 18–55 years, with BMI 18.5–29.9 kg/m^2^, and weight between 55 and 100 kg were eligible for inclusion. Written informed consent was provided by all participants.

Exclusion criteria included history of drug, alcohol, and/or tobacco/nicotine abuse; use of any prescription medication (with exception of hormone replacement therapy for females) within 14 days prior to Day 1; use of any over-the-counter medication, including herbal products and vitamins, within the 7 days prior to Day 1; use of antacids, H2 receptor blockers or proton pump inhibitors 3 days prior to Day 1; history of hypersensitivity or allergic reaction to ceftibuten, any of the excipients used in the formulation, or any beta-lactam antibiotics; females pregnant or lactating; calculated creatinine clearance <80 mL/min (Cockcroft–Gault method) at screening or Day 1; clinically significant abnormalities on laboratory values at screening or Day 1.

### Data collection

The participant data collected before and during the study included age, sex, height, weight, body mass index, electrocardiogram monitoring, vital signs, hematology, coagulation, serum chemistry, urinalysis, and serology.

### Blood and urine sampling

Blood samples were collected pre-dose and 1, 1.5, 2, 3, 4, 6, 8, 12, and 24 h post-dose on Days 1 and 10, and pre-dose and 1, 1.5, 2, 3, 4, 6, 8, and 12 h after the first dose on Day 4. A 5-min deviation window was permitted for the nominal times 0–4 h, and 10 min for the nominal times 4–24 h. After collection, the blood samples were centrifuged at 3,000 rpm for 10 min, and the resultant plasma was frozen at −20°C until analysis.

Urine samples were collected pre-dose and 0–4, 4–8, 8–12, 12–24, and 24–48 h post-dose on Days 1 and 10, and pre-dose and 0–4, 4–8, and 8–12 post-dose on Day 4. Subjects were encouraged to void completely within 15 min prior to dosing, and at the end of each collection interval. A 20-min deviation window was permitted. Urine was refrigerated during the collection intervals. At the end of each interval, the total urine volume was measured. The volumes were recorded, and the samples were frozen at −20°C until analysis.

### Sample analysis

The plasma and urine concentrations of *cis-* and *trans-*ceftibuten were assayed using a validated high-performance liquid chromatography method coupled with ultraviolet detection (HPLC-UV) (Microconstants, San Diego, CA, USA) (24). The HPLC column used was a Restek Ultra Biphenyl, 5 µm, 150 × 3.0 mm. UV detection was set at 254 nm. *Cis-* and *trans-*ceftibuten retention times were 4.5 and 5.4 min, respectively. The internal standard was cefdinir.

Acetonitrile (500 µL) was added to the plasma samples (500 µL), vortex-mixed for 10 s and centrifuged at 3,000 × *g* for 10 min. Afterward, 800 µL of the supernatant was obtained and added to 5 mL of dichloromethane, vortex-mixed for 10 s, and centrifuged again. The aqueous solution was separated, and 50 µL was injected into the chromatographic system. For the plasma assay, the mobile phase consisted of ammonium acetate (3.85 g/L in water and 2 mL of triethylamine, adjusted to pH four with formic acid)/acetonitrile (95.5/4.5) at a flow rate of 1.75 mL/min.

The correlation coefficients of the plasma calibration curves were not less than 0.996 for *cis*-ceftibuten and 0.997 for *trans*-ceftibuten and were linear across the range of 0.125–50.0 mg/L. For *cis-*ceftibuten in plasma, the intra- and inter-day accuracy was −3.20% to 4.25% and −0.75% to 0.67%, respectively; precision was 0.47%–5.63% and 1.60%–3.44%, respectively. For *trans-*ceftibuten in plasma, the intra- and inter-day accuracy was −6.93% to 7.33% and −1.33% to 1.25%, respectively; precision was 0.71%–8.48% and 4.05%–5.21%, respectively.

Urine samples were diluted (1/10–1/40) with purified water. A volume of 50 µL was injected into the chromatographic system. For the urine assay, the mobile phase consisted of Na_2_HPO_4_ (17.9 g/L in water, adjusted to pH 7 with H_3_PO_4_ 10%)/acetonitrile (97.5/2.5) at a flow rate of 1 mL/min.

The correlation coefficients of the urine calibration curves were not less than 0.998 for *cis*-ceftibuten and *trans*-ceftibuten and were linear across the range of 0.3125–125 mg/L. For *cis-*ceftibuten in urine, the intra- and inter-day accuracy was −2.51% to 2.00% and 0.00%–0.59% respectively; precision was 0.49%–5.05% and 1.65%–2.52%, respectively. For *trans-*ceftibuten in urine, the intra- and inter-day accuracy was −6.13% to 4.00% and −1.12% to 2.00%, respectively; precision was 0.38%–8.80% and 3.16%–3.73%, respectively.

### Pharmacokinetics

PK parameters were calculated from the individual plasma concentrations, and urine concentrations and volumes, by non-compartmental analysis using Phoenix WinNonlin version 8.2.0.4383 (Certara, Princeton, NJ, USA). Summary statistics (*N*, mean, and SD) were tabulated by dosing group and day for each of the PK parameters. Plasma PK parameters *C*_max_, *T*_max_, AUC_0–12_, AUC_0–INF_, *t*_1/2_, Vz/*F*, and CL/*F* were obtained for *cis-* and *trans*-ceftibuten. Urine PK parameters Ae, fe, and CL_R_ were obtained for *cis*-, *trans*-, and total ceftibuten to characterize its elimination.

All concentration values below the quantification limit were treated as missing. A terminal phase could not be defined for some concentration-time curves, and consequently no value for AUC_0–INF_, *t*_1/2_, Vz/*F*, CL/*F*, and CL_R_ were calculated.

### Dose proportionality

Dose proportionality for increasing oral dose and plasma pharmacokinetic parameters was examined graphically for *cis-* and *trans-*ceftibuten using AUC_0–12_ values on Days 1, 4, and 10 using linear regression. The equation for linear regression model fit was AUC = *β* × dose + *μ* where *β* represents the slope of the regression model and *µ* is the intercept. If the slope for each day is greater than 0, then some evidence of proportionality is assumed.

Dose proportionality was also assessed statistically using the exposure parameters of Cmax, AUC_0–12_ and AUC_0–INF_. Each subjects' parameter values from the three sampling days were normalized to 400 mg and an ANOVA was performed. If the ANOVA achieved statistically significant results (*P* ˂ 0.05), post hoc tests were performed: Tukey Kramer for normally distributed data and Kruskall–Wallis for non-normally distributed data. Normality was assessed with the Shapiro–Wilk test.

### Accumulation in plasma exposure

Accumulation of *cis*- and *trans-*ceftibuten going from a single dose to steady state with repeated administrations was calculated as the ratio of Day 10 AUC_0–12_/Day 4 AUC_0–12_.

### Safety and tolerability

AE monitoring was conducted daily during the trial. An AE was defined as any unfavorable and unintended sign, symptom, or disease temporally associated with use of ceftibuten. Each AE was assessed for its relationship to drug treatment (not related, unlikely, possible, and probable) with signs or symptoms graded on a 5-point severity scale (mild, moderate, severe, life-threatening, and death). Serious AE monitoring was conducted as part of the AE monitoring.
